# Osteolysis of unknown origin: a case report

**DOI:** 10.1186/s12903-015-0158-8

**Published:** 2015-12-30

**Authors:** Matthias Guido Wiesli, Katrin E. Hostettler, Michael Tamm, Claude Jaquiéry

**Affiliations:** Clinic for Craniomaxillofacial and Oral Surgery, University Hospital Basel, Spitalstrasse 21 4031, Basel, Switzerland; Clinics of Respiratory Medicine, University Hospital Basel, Petersgraben 4 4031, Basel, Switzerland

**Keywords:** Sarcoidosis, Bone lesion, Jaw angle

## Abstract

**Background:**

Sarcoidosis is a granulomatous disease that may affect any organ of the body. The most frequent loci of manifestation are the lungs. However, there are individual cases where bones are affected. The literature describes cases in which swelling or fistula were the first findings of a bone lesion. This is the first case reporting an osteolysis in both angles of the mandibles which led to the diagnosis of sarcoidosis with multi-organ involvement.

**Case presentation:**

The authors present a 74 years old European female patient without previous diagnosis of sarcoidosis who presented with pain in the area of the jaw angles. There were no further clinical symptoms. Bone biopsy following radiological investigation demonstrated non-caseating granulomas consistent with sarcoidosis of the bone. Further evaluation confirmed multi-organ disease with involvement of lungs, intrathoracic lymph nodes, and the central nervous system.

**Conclusion:**

This case report shows that diagnosis of a severe disease can be missed if systematic clinical signs are not given. Furthermore, an accurate anamnesis and examination is required to receive an early diagnosis which often needs an interdisciplinary approach.

## Background

Sarcoidosis is a chronic granulomatous disease of unknown origin with clinical manifestations being highly variable between affected patients. Sarcoidosis was first described by the surgeon and dermatologist Jonathan Hutchinson in London more than 100 years ago [1, 2]. An exaggerated immune response without distinct stimulus is characteristic for sarcoidosis. So far, no specific environment trigger could be identified which leads to this obscure disease. Newman et al. suggest that multiple environment and occupational agents could induce hyperactivity of the human immune system [3]. However, sarcoidosis is actually described as idiopathic systemic disease affecting people of different ethnic backgrounds and races. All ages are involved while the highest incidence is among people between 20 to 39 years of age [4]. Northern Europe represents the highest annual incidence (5 to 40 of 100’000) while Japan has a lower range of 1 to 2 cases of 100’000 per year [5]. Localisation of the disease and clinical manifestations are highly variable between affected patients. The lungs are most commonly affected with more than 90 % of the patients having pulmonary involvement [6]. Extra-pulmonary organ manifestation such as eyes, liver, peripheral lymph nodes and skin represent a lower prevalence with approximately 10 % to 30 % of patients affected [7]. The incidence of bone involvement differs from less than 5 % up to 13 % [7, 8]. Long bones, the axial skeleton as well as hands and feet can be affected by the disease. Most patients with bone involvement are asymptomatic. Bony lesions usually occur bilateral and are characterized by an osteolytic zone, while the cortical part remains intact. Different modalities are used to diagnose osseous involvement, such as conventional radiography, computed tomography- (CT) or magnetic resonance imaging (MRI), technetium-99 m bone scintigraphy, and positron emission -computed tomography (PET-CT) [8].

The clinical appearance is highly variable, involving sustained cough, erythema nodosum, periarticular inflammation, fever and occurring fatigue. The diagnosis is based on three pillars: (1) clinical assessment, (2) radiographic signs and (3) histological evidence of non-caseating granulomas [7]. A biopsy is necessary to confirm the diagnosis of a sarcoidosis. Therapy consists of immunosuppressive treatment with oral glucocorticoids being the standard first-line treatment. Steroid-sparing agents, such as methotrexate, azathioprine, or mycophenolate mofetil have been used for treatment of sarcoidosis.

## Case presentation

**Fig. 1:**
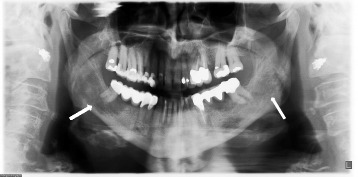
Panoramic X-ray. The arrows on the panoramic X-ray show osteolytic zones on the right and left angle of the lower jaw

A 74 year old European female patient approached her dentist due to pain in the right lower jaw. A panoramic X-ray was performed, presenting an osteolysis of unknown origin in the mandible on both sides, right more than left (Fig. 1). The patient was then referred to the clinic of cranio-maxillo-facial surgery for further evaluation. She reported pain in the area of the right angle of the mandible for several months; additionally, pain in the right calcaneus and in both forearms has been noted. She denied fever and weight loss, but reported cough and dyspnoea on exertion. Furthermore, a sicca-symptomatic in both eyes and the oral cavity was present. The patient was diagnosed with hypertensive cardiac disease, rhytmogene cardiopathy, hyperlipidaemia, cutaneous psoriasis with psoriasis arthritis, and lumbar vertebral syndrome several years before. Her home medication included pantoprazol, candesartane, acetylsalicylate, spironolactone, and simvastatine.

At the day of first presentation there were no signs for locale infection on the oral and extraoral examination, and no palpable lymph nodes in the cervical region. Only the tooth 47 showed percussion sensitivity and was not sensitive to cold. There were no abnormal laboratory findings, specifically normal C-reactive protein, serum calcium and alkaline phosphatase.

**Fig. 2:**
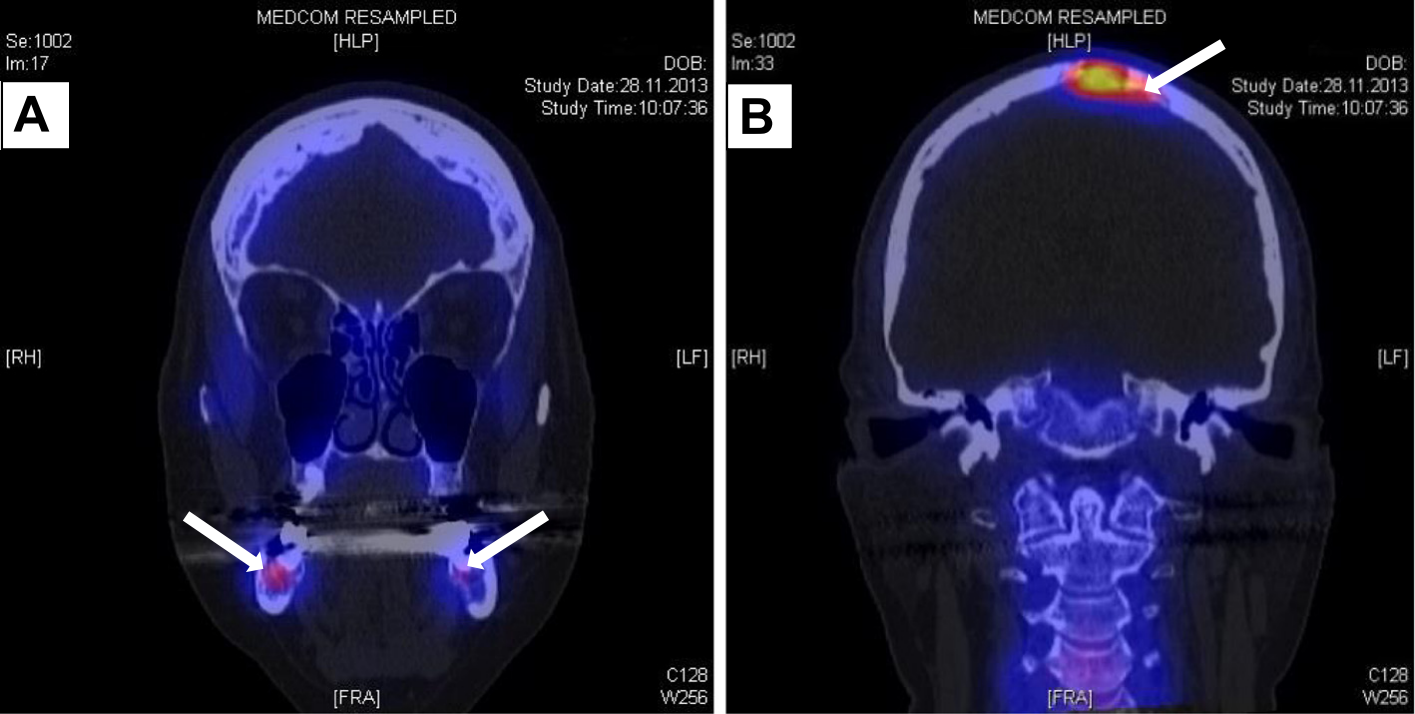
SPECT-CT imaging of the head. The coronal view presents increased bone metabolism in the area of both jaw angles **a** and in the area of the vertex **b** which is indicated with white arrows

**Fig. 3:**
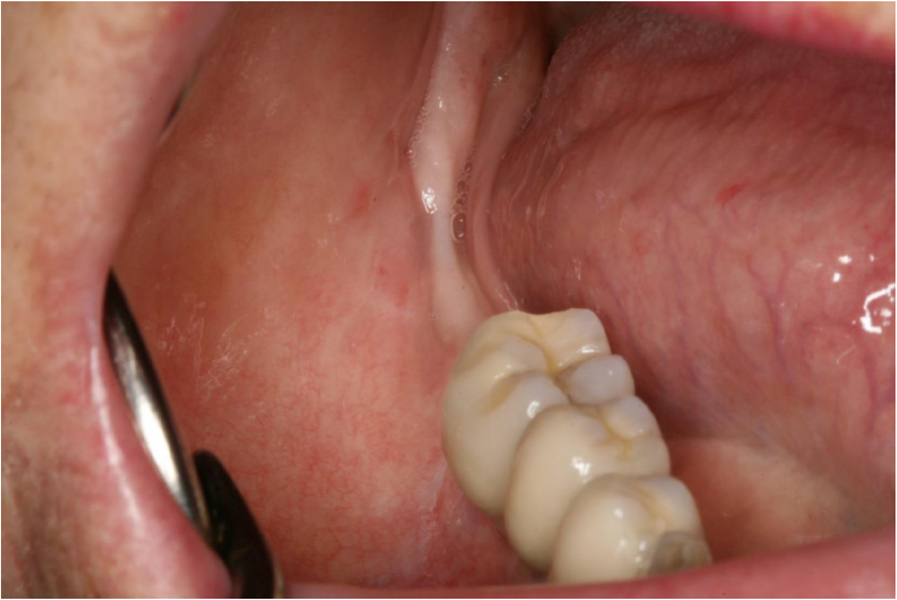
Clinical situation of the right lower jaw. This photograph shows the intraoral situation after removing the second lower molar. The soft tissue healed without any complication

A computed tomography with 3D reconstruction of the osteolysis was performed. The radiography showed loosening of cancellous bone with extension of the mandibular canal and partially interrupted cortical bone on the right side. The findings were suspicious for osteomyelitis or a malignant lesion. Additional Single Photon Emission Computed Tomography (SPECT-CT) examination was completed presenting increased bone metabolism in the area of the vertex and the jaw angles of both sides, right more than left (Fig. 2). Further increased metabolism was detected in both forearms and in the right calcaneus. A biopsy of the alveolar process of the lower jaw was carried out for further investigation of the unknown osteolysis together with the removal of the second lower molar (Fig. 3). Histology revealed chronic granulomatous inflammation with non-caseating epitheloid granuloma, consistent with sarcoidosis. Ziehl-Neelsen’s staining was negative and mycobacterial culture showed no growth. Fungal and other bacterial infections were excluded. To confirm the suspicion of sarcoidosis further examinations were initiated: The CT-Scan of the lung showed enlarged intrathoracic lymph nodes, and pulmonary function testing revealed a restriction of diffusion capacity of carbon monoxide. Based on a recently developed vertigo a MRI scan of the brain and the spinal cord was performed, showing a suspicious lesion in the area of the vertex compatible with neuro-sarcoidosis. Levels of interleukin-2-receptor and angiotensin-converting enzyme were in the range of normal. Finally, biopsy of an additional skin lesion in the area of the medial canthus of the left eye histologically confirmed the diagnosis of sarcoidosis.

Due to relevant multi-organ disease with symptomatic bone involvement and strong suspicion of neuro-sarcoidosis immunosuppressive therapy with azathioprine was installed. A follow-up SPECT-CT scan after six months of treatment revealed regressive bone metabolism of all previously described lesions, and no new lesions were detected. The patient was free of pain and the immunosuppressive treatment was well tolerated.

A couple of differential diagnosis should be considered when evaluating intrabony lesions of the jaws, in particular of the lower jaw: osteomyelitis, metastatic tumors, multiple myeloma, Langerhans histiocytosis. All these findings may present a similar radiographic image and could be confounded with a sarcoidotic lesion and had therefore to be considered in our patient.

There are a few cases in literature reporting sarcoidosis with manifestation in the lower jaw. Monasebian et al. described the diagnosis of sarcoidosis by means of a recurrent swelling of the chin in 1997 [9]. Swelling followed by tenderness were the first clinical signs of systemic sarcoidosis. The lesion was excised and histologically investigated. It showed a florid granulomatous inflammation. Suresh et al. presented a case where the bone lesion in the lower jaw occurred one year after sarcoidosis was diagnosed [10]. Here, loose teeth were the consequence of the destroyed bone by osteolysis. Authors postulated not to confound this clinical sign with an aggressive periodontitis. A different case was reported by Grimaldi et al. [11]. They revealed that the first sign of sarcoidosis was a fistula in the area of the lower left canine. The panoramic X-ray showed an intra-bony lesion within the correspondent area. However, the tooth was not mobile and there were no other clinical signs.

Our case differs from aforementioned studies as it began with an osteolysis of unknown origin in the mandible on both sides. To our knowledge there is no scientific literature about the onset of sarcoidosis on the lower jaw.

Systematic signs could be even missed in some cases. This fact may not lead astray. We suggest considering a chronic granulomatous disease in cases of intra-bony lesions. Each obscure bone lesion has to be biopsied to get the accurate diagnosis followed by the adequate therapy.

## Conclusions

The first symptoms of multi-organ sarcoidosis can be variable and symptomatic bone involvement is a rare presentation of this disease. Histologic confirmation is mandatory, followed by further diagnostic assessments with regard to other organ involvement. This case is of utmost clinical importance as it demonstrates that an accurate anamnesis and clinical and radiological examination are required for an early diagnosis of this rare disease.

### Consent

Written informed consent was obtained from the patient for publication of this Case report and any accompanying images. A copy of the written consent is available for review by the Editor of this journal.
